# Efficacy and Safety of the Triple Therapy Containing Ilaprazole, Levofloxacin, and Amoxicillin as First-Line Treatment in *Helicobacter pylori* Infections

**DOI:** 10.1155/2017/1654907

**Published:** 2017-04-28

**Authors:** Hyo Jun Ahn, Dong Pil Kim, Myeong Su Chu, Hyeon Jeong Yun, Seok-Hwan Kim, Seung Woo Lee, Dong Soo Lee

**Affiliations:** Division of Gastroenterology, Department of Internal Medicine, College of Medicine, Daejeon St. Mary's Hospital, The Catholic University of Korea, Daejeon 34943, Republic of Korea

## Abstract

*Background and Aims.* To establish the efficacy and safety of ilaprazole, levofloxacin, and amoxicillin as a first-line eradication treatment for *Helicobacter pylori*. *Methods.* Patients with gastric ulcer, duodenal ulcer, or gastritis, as detected by esophagogastroduodenoscopy with confirmed *H. pylori* infection between September 2014 and November 2015, were enrolled in the study. All participants received ilaprazole (10 mg bid), levofloxacin (500 mg bid), and amoxicillin (1000 mg bid) for 10 days. *H. pylori* eradication was confirmed by a ^13^C-urea breath test at 6–8 weeks after the end of treatment. *Results.* Of 84 patients included in the analysis, the eradication rate was 88.8% in the per protocol group (*n* = 80). Demographic factors such as age, gender, body mass index (BMI), alcohol, smoking, hypertension, diabetes mellitus, and peptic ulcer did not affect the eradication rate. However, multivariate analysis showed that overweight patients and patients with cerebrovascular accident (CVA) had a significantly lower eradication rate than patients with normal BMI and without CVA. Laboratory test results did not change significantly after treatment. A total of six (7.5%) patients developed eight adverse reactions. *Conclusions.* A 10-day triple therapy containing ilaprazole, levofloxacin, and amoxicillin is a safe alternative first-line eradication treatment for *H. pylori*.

## 1. Introduction

*Helicobacter pylori* colonize in the stomach and cause various diseases such as peptic ulcers, gastric cancer, and gastric mucosa-associated lymphoid tissue (MALT) lymphoma. Furthermore, *H. pylori* infections cause extragastric diseases such as iron-deficiency anemia, vitamin B12 deficiency, and idiopathic thrombocytopenic purpura. Current clinical guidelines recommend a clarithromycin-containing standard triple therapy (STT) as the first-line treatment and a bismuth-containing quadruple therapy (BCQT) or levofloxacin-based therapy (LBT) as the second-line treatment after failure of the STT [1, 2]. However, due to increasing clarithromycin resistance [3], the eradication rate using the STT has decreased and ranges from 61.8 to 77%. The Maastricht IV/Florence consensus report recommends that STT should not be used in regions with a clarithromycin resistance rate of more than 15–20%. The second-line treatment, BCQT, has poor compliance due to side effects and complicated administration. For this reason, extensive research has been conducted to find alternative therapies such as sequential therapy, concomitant therapy, LBT, and rifabutin-based therapy to replace STT. LBT has been mainly used as a rescue therapy because quinolone resistance is a concern. However, LBT as a first-line therapy has remained controversial. Clinical trials investigating LBT as a first-line therapy have reported eradication rates from 72 to 96% [4, 5].

Ilaprazole is a new-generation PPI that is well known for its extended plasma half-life and metabolism, which is not significantly influenced by CYP2C19 [6–8].

In this study, we evaluate the efficacy and safety of ilaprazole (10 mg bid), levofloxacin (500 mg bid), and amoxicillin (1000 mg bid) as a first-line treatment for *H. pylori* infection.

## 2. Materials and Methods

### 2.1. Study Population

In this single-center prospective study, we included patients with gastric ulcer, duodenal ulcer (including scar), and gastritis with confirmed *H. pylori* infection by histology, rapid urease test, or ^13^C-urea breath test (UBT). Patients were recruited from the outpatient gastroenterology department in Daejeon St. Mary's Hospital between September 2014 and November 2015. This study was approved by the Institutional Review Board of the Catholic University of Korea and registered at ClinicalTrials.gov (NCT02352701). All participants agreed to take part in this clinical study and provided informed consent. Exclusion criteria included age under 20 years; allergy or hypersensitivity to the test drugs; pregnancy, breast feeding, childbearing age, and not using appropriate contraception; uncontrolled diabetes mellitus (DM) or hypertension (HTN); drug or alcohol abuse; history of malignancy within the last 5 years (excluding those who underwent endoscopic curative resection for gastric dysplasia or early gastric cancer); history of surgery such as esophagectomy or gastrectomy; hereditary diseases such as galactose intolerance, lactase deficiency, and glucose-galactose malabsorption; and current participation in another clinical trial. Furthermore, we excluded subjects who had the following laboratory abnormalities: (1) ≥1.5 times the upper limit of normal in terms of total bilirubin and creatinine (Cr) levels and (2) ≥2 times the upper limit of normal in terms of aspartate transaminase (AST), alanine transaminase (ALT), alkaline phosphatase (ALP), and blood urea nitrogen (BUN) levels. We dropped out subjects who (1) withdrew agreement to participate, (2) took a drug that adversely affected the efficacy and safety of the test drugs during the study, and (3) had less than 80% drug compliance. We withdrew subjects who had critical adverse reactions that required hospitalization and laboratory abnormalities over 1.5 times that of baseline values, regardless of whether or not the cause was the test drugs.

### 2.2. Study Design

This study used a single-center prospective observational clinical trial design to evaluate the efficacy and safety of an ilaprazole-based triple therapy as a first-line therapy for *H. pylori* treatment. All subjects received ilaprazole (10 mg bid), levofloxacin (500 mg bid), and amoxicillin (1000 mg bid) for 10 days and understood that they were required to visit our laboratory or outpatient clinic following completion of the study protocol. During the screening phase of the study, informed consent was obtained. We also obtained demographic data, performed a physical examination and esophagogastroduodenoscopy (EGD), obtained baseline laboratory values, and confirmed *H. pylori* infection by histology, ^13^C-UBT, or rapid urease test in subjects with gastric ulcer, duodenal ulcer, or gastritis. Two weeks after screening, we assessed participants with respect to the inclusion and exclusion criteria and prescribed the test drugs. After completion of the eradication therapy, we assessed adverse reactions, drug compliance, and laboratory test results.

### 2.3. Follow-Up EGD and ^13^C-Urea Breath Test (UBT)

After 6–8 weeks of the eradication therapy, we performed EGD and assessed *H. pylori* status by ^13^C-UBT. Ulcer healing was considered successful if an ulcer in active or healing stage resolved to scar stage. To perform UBT, patients swallowed ^13^C-urea-labeled compositions (Helifinder kit; Medichems, Seoul, South Korea). After 20 min, the ^13^CO^2^/^12^CO^2^ ratio was determined with a HeliView analyzer (Medichems) and was considered positive if the delta (0–20 min) ratio of ^13^CO^2^/^12^CO^2^ was more than 2 (positive UBT).

### 2.4. Drug Compliance and Adverse Reactions

Drug compliance and adverse reactions were assessed during scheduled visits and phone calls. We defined significant drug compliance as intake of more than 80% of the test drug and planned to exclude participants with less than 80% compliance. Adverse reactions were graded based on the need for treatment and hospitalization.

### 2.5. Endpoints

The primary endpoint of the study was an *H. pylori* eradication rate. The secondary endpoints were safe during study period and ulcer healing rate within the PUD group.

### 2.6. Statistical Analysis

Categorical data were compared using the X^2^ test or Fisher's exact test, and continuous data were compared using Student's *t*-test. Multiple logistic regression analysis was performed for evaluation of major risk factors affecting the eradication rate, and 95% confidence intervals (CIs) were provided for all comparisons. A *p* value of <0.05 was considered statistically significant. Statistical analyses were performed using SPSS for Windows software (ver. 22.0; IBM Corp., Armonk, NY, USA).

## 3. Results

### 3.1. Baseline Characteristics of the Subjects

Baseline characteristics of the subjects are shown in Table 1. In total, 84 subjects were included in the study and 4 subjects were dropped out because their drug compliance was below 80%. Therefore, 80 subjects completed the study, of whom 32 (40%) were male and 48 (60%) were female. In the per protocol (PP) group, the age of the subjects ranged from 41 to 73 years, and the mean age was 57.41 ± 7.73 years. The body mass index (BMI) of the patients ranged from 15.97 to 30.85, and the mean BMI was 24.27 ± 2.85. Of the subjects, 11 (13.8%) were current smokers, 18 (22.5%) had a history of alcohol ingestion, 8 (10%) had a history of DM, 29 (36.3%) had a history of HTN, and 6 (7.5%) had a history of liver disease (3 subjects were in an inactive hepatitis B virus (HBV) carrier state, 2 had compensated liver cirrhosis due to chronic hepatitis B, and 1 had chronic hepatitis C). In total, 18 (22.5%) subjects had the following histories beyond those mentioned above (number of subjects is shown in parentheses): Addison's disease (1), benign prostatic hyperplasia (2), hyperlipidemia (3), psoriasis (1), rheumatoid arthritis (1), thyroid cancer (8), ovarian cancer (1), and gastric dysplasia with endoscopic mucosal resection (1). Of the subjects, nine (11.25%) took nonsteroidal anti-inflammatory drugs (NSAIDs) and antithrombotic agents, six took aspirin, two took clopidogrel, and one took NSAIDs only. Twenty (25%) patients were peptic ulcer disease (PUD) patients, and the other 60 (75%) patients were gastritis patients. There were no abnormal findings on baseline laboratory tests (Table 2).

### 3.2. Laboratory Changes after Eradication

There were no significant laboratory changes after *H. pylori* eradication. Although the albumin level increased from 4.507 ± 0.300 to 4.506 ± 0.299 (*p* = 0.025) and the creatinine level decreased from 0.781 ± 0.173 to 0.755 ± 0.163 (*p* = 0.008), after eradication, these changes were not clinically significant. Also, *H. pylori* eradication had no effect on the glucose and lipid profile (Table 2).

### 3.3. Eradication Rate

The eradication rate was 84.5% (95% CI: 65.8–106.5%) in the intention-to-treat (ITT) group and 88.8% (95% CI: 69.3–112.0%) in the per protocol (PP) group. Demographic factors including age, gender, alcohol consumption, smoking status, and presence of HTN, DM, and liver disease (such as chronic viral hepatitis) did not affect the eradication rate. The presence or absence of peptic ulcer also did not affect the eradication rate. Subjects were categorized as underweight (BMI < 18.5), normal weight (BMI = 18.5–24.99), overweight (BMI = 25.00–29.99), or obese (BMI ≥ 30). There were few underweight (*n* = 1) and obese (*n* = 1) subjects. In subgroup analysis, the eradication rate of subjects with normal BMI was higher than that of overweight subjects (95.6% versus 78.8%; *p* = 0.032) on PP analysis. However, there was no significant difference on ITT analysis. Among the three subjects who had a history of cerebrovascular accident (CVA), the eradication rate was 33.3%. CVA was a demographic factor that led to a decrease in the eradication rate, but NSAID or antithrombotic agent use had no effect (Table 3). In multiple regression analysis, overweight subjects had a significantly lower eradication rate than normal weight subjects (odds ratio (OR) = 5.929, 95% CI: 1.047–33.585; *p* = 0.076). Subjects with CVA also had a lower eradication rate on multiple regression analysis (OR = 18.571, 95% CI: 1.488–231.719; *p* = 0.023). The presence of DM, HTN, liver disease, peptic ulcer disease, drug history, alcohol, and smoking did not affect the eradication rate (Table 4).

In the PUD subgroup (*n* = 20) in PP analysis, the ulcer healing rate was 90%.

### 3.4. Compliance and Adverse Events

In total, 80 subjects completed the study, all of whom were more than 80% compliant. The mean compliance rate was 99.6%. Six subjects developed eight adverse reactions (Table 5). The incidence of adverse reactions was 7.5% and the frequency was 10%. The most common side effects were nausea, vomiting, and dizziness. The severity of the adverse events ranged from grade 1 to grade 2 and no subjects required hospitalization.

## 4. Discussion

The present study showed that a 10-day ilaprazole-based regimen consisting of ilaprazole, levofloxacin, and amoxicillin had a relatively high eradication rate and favorable safety compared with STT. Adverse events and laboratory changes were not significant during the treatment period, and CVA and being overweight were significant risk factors for low eradication rate.

The clarithromycin resistance rate has increased from 9% to 17.6% in Europe and from 17.2% to 37.3% in South Korea [9–12]. The Maastricht IV/Florence consensus report recommends that STT be avoided in regions with a clarithromycin resistance rate greater than 15–20% and recommends BCQT as an alternative first-line therapy. However, BCQT has poor compliance due to side effects and complicated administration [2]. Therefore, therapies other than the current STT are needed. Alternative approaches include change of regimen and longer treatment.

Levofloxacin is a fluoroquinolone antibacterial agent that inhibits bacterial DNA topoisomerase II. LBTs have been used primarily as salvage therapies after failure of first-line therapy. Recently, many studies have investigated the use of LBT as a first-line therapy. The mean eradication rate with LBT as a first-line or rescue therapy was reported to be approximately 80% based on a meta-analysis [13, 14]. As a salvage therapy, 10 days of LBT was effective than 7 days of LBT according to meta-analysis [13]. Moreover, LBT had less adverse effects than BCQT [13].

The present study showed that the *H. pylori* eradication rate after 10 days of LBT using ilaprazole was 88.8%. This was much higher than the eradication rate after 10 days of LBT using omeprazole and lansoprazole (72–83%) [13, 15, 16] and was comparable with that of LBT using esomeprazole (87–96%) [17, 18]. This suggests that ilaprazole may have unique properties and better pharmacokinetic and pharmacodynamic profiles compared with other PPIs. CYP2C19 polymorphisms have been thought to affect the *H. pylori* eradication rate. Proton pump inhibitors (PPIs) are metabolized by the hepatic cytochrome p450 (CYP) system, primarily CYP2C19. In patients carrying the poor metabolizers (PM) genotype, the metabolism of PPIs is much slower, resulting in a PPI bioavailability that is more than 20 times that of the bioavailability in homozygous extensive metabolizers (HomEM) [19].

In South Korea, recent *H. pylori* eradication rates have been reported to be 75.5–78.7%. This is mainly due to a high clarithromycin-resistance rate; however, it is also possible that the cause is a CYP2C19 polymorphism [20]. Ilaprazole is primarily metabolized by CYP3A4 and is not influenced by CYP2C19 genetic polymorphisms [21]. In addition, ilaprazole has a long half-life, which leads to a longer period of gastric acid suppression and higher ulcer healing rates compared with other PPIs. In a recent multicenter, randomized double-blind controlled trials in China, subjects taking ilaprazole had significantly higher ulcer healing rates than those taking omeprazole, especially those with the EM genotype [22]. In this study, in the PUD subgroup (*n* = 20), the ulcer healing rate was 90%, consistent with results from previous studies.

Some recent reports revealed that risk factors for eradication failure include high bacterial load, strain type, high gastric acidity, clarithromycin resistance, and low compliance [20, 23]. In the current study, demographic factors such as alcohol, smoking, HTN, DM, and liver disease (such as chronic viral hepatitis) did not affect the eradication rate. Furthermore, multivariate analysis showed that age, gender, NSAID use, and antithrombotic drug use did not affect the eradication rate. Overweight and CVA were risk factors for treatment failure on our multivariate analyses. With regard to BMI, there was a report claiming that overweight patients showed a significantly lower *H. pylori* eradication rate than controls [24], while there was a report that revealed opposite results [20]. Future research should be conducted to determine if there is a correlation between the *H. pylori* eradication rate and BMI. *H. pylori* infection may increase the risk for ischemic stroke. In a recent meta-analysis, a relative risk of 2.42 was obtained for stroke and *H. pylori* infection [25]; therefore, *H. pylori* eradication may prevent stroke [26]. However, there is no report that *H. pylori* eradication rate is associated with CVA. Further investigations are needed to determine if there is a causal relationship between CVA and *H. pylori* eradication.

## 5. Conclusions

Our ilaprazole, levofloxacin, and amoxicillin treatment regimen is a safe and a valuable alternative first-line therapy for *H. pylori* eradication. The safety profile of this therapy was favorable, and there were no significant laboratory changes during the treatment period. Although CVA and overweight were risk factors for eradication failure in our study, further investigation is needed to confirm this.

## Figures and Tables

**Table 1:** Baseline characteristics of the participants.

Characteristics	ITT group	PP group
Number	84	80
Mean age, years ± SD	57.37 ± 7.62	57.41 ± 7.73
Male, *n* (%)	34 (40.5)	32 (40.0)
Height, m ± SD	1.62 ± 0.08	1.61 ± 0.73
Weight, kg ± SD	63.6 ± 9.99	63.78 ± 10.18
BMI, kg/m^2^ ± SD	24.23 ± 2.81	24.27 ± 2.85
Alcohol, *n* (%)	18 (21.4)	18 (22.5)
Smoking, *n* (%)	11 (13.1)	11 (13.8)
DM, *n* (%)	8 (9.5)	8 (10.0)
HTN, *n* (%)	29 (34.5)	29 (36.3)
CVA, *n* (%)	3 (3.6)	3 (3.8)
Liver disease, *n* (%)	6 (7.1)	6 (7.5)
Others, *n* (%)	20 (23.8)	18 (22.5)
NSAIDS or antithrombotic agent, *n* (%)	9 (10.7)	9 (11.25)
With peptic ulcer, *n* (%)	21 (25.0)	20 (25.0)

ITT: intention-to-treat; PP: per protocol; SD: standard deviation; BMI: body mass index; DM: diabetes mellitus; HTN: hypertension; CVA: cerebrovascular accident; NSAIDs: nonsteroidal anti-inflammatory drugs.

**Table 2:** Comparison of laboratory findings before and after treatment.

	Before treatment	After treatment	*p* value
White blood cell count (10^3^/mm^3^)	5.849 ± 1.515	5.891 ± 1.424	0.753
Hemoglobin (g/dL)	14.040 ± 1.288	13.928 ± 1.2409	0.123
Platelet count (10^3^/mm^3^)	228.33 ± 42.698	222.11 ± 41.633	0.054
Total protein (g/dL)	7.224 ± 0.386	7.230 ± 0.326	0.868
Albumin (g/dL)	4.506 ± 0.299	4.578 ± 0.268	0.025^∗^
Total bilirubin (mg/dL)	0.773 ± 0.448	0.750 ± 0.552	0.308
Aspartate transaminase (IU/L)	22.33 ± 4.862	22.70 ± 6.053	0.580
Alanine transaminase (IU/L)	21.95 ± 8.968	22.78 ± 10.624	0.472
Alkaline phosphatase (IU/L)	61.105 ± 16.448	62.150 ± 16.613	0.503
*γ*-Glutamyl transpeptidase (IU/L)	22.09 ± 15.693	22.70 ± 13.756	0.063
Blood urea nitrogen (mg/dL)	13.620 ± 3.475	13.694 ± 3.376	0.863
Creatinine (mg/dL)	0.781 ± 0.173	0.755 ± 0.163	0.008^∗^
Glucose (mg/dL)	101.31 ± 13.392	99.95 ± 12.849	0.139
Total cholesterol (mg/dL)	200.14 ± 34.964	195.49 ± 30.820	0.185
Triglyceride (mg/dL)	133.56 ± 80.728	128.68 ± 80.598	0.462

^∗^ *P* < 0.05.

**Table 3:** Subgroup analysis of eradication rate.

Group (*n*)	Intention-to-treat analysis				Per protocol analysis			
	Eradication rate, *n* (%)	Relative risk	95% CI	*p* value	Eradication rate, *n* (%)	Relative risk	95% CI	*p* value
Total	**75 (84.5)**				**71 (88.8)**			
Age				0.947				1.000
<60 years old	43 (84.3)	1.035	0.370–2.894		43 (89.6)	1.024	0.870–1.205	
≥60 years old	28 (84.8)	0.994	0.825–1.197		28 (87.5)	0.833	0.242–2.869	
Gender				0.650				1.000
Male	28 (82.4)	0.958	0.791–1.160		28 (87.5)	0.977	0.830–1.149	
Female	43 (86.0)	1.261	0.464–3.425		43 (89.6)	1.200	0.349–4.132	
BMI				0.064				0.032^∗^
Normal BMI	43 (91.5)	1.232	0.995–1.525		43 (95.6)	1.213	1.005–1.464	
Overweight BMI	26 (74.3)	0.331	0.111–0.988		26 (78.8)	0.210	0.046–0.945	
Alcohol				0.281				0.676
Drinker	17 (94.4)	3.279	0.455–23.522		16 (94.4)	2.323	0.311–17.361	
Nondrinker	54 (81.8)	0.866	0.738–1.016		55 (87.1)	0.922	0.796–1.069	
Smoking				1.000				1.000
Smoker	10 (90.9)	1.268	0.260–12.570		10 (90.9)	1.275	0.176–9.229	
Nonsmoker	61 (83.6)	0.919	0.743–1.137		61 (88.4)	0.972	0.792–1.194	
NSAIDs or antithrombotic agents				0.603				0.220
Yes	6 (75)	0.579	0.155–2.165		6 (75)	0.389	0.097–1.564	
No	65 (85.5)	1.140	0.756–1.719		65 (90.3)	1.204	0.801–1.809	
HTN				0.203				0.476
Yes	27 (93.1)	2.900	0.689–12.215		27 (93.1)	1.990	0.442–8.954	
No	44 (80.0)	0.859	0.728–1.014		44 (86.3)	0.927	0.799–1.074	
DM				1.000				1.000
Yes	7 (87.5)	1.263	0.188–8.492		7 (87.5)	0.889	0.127–6.225	
No	64 (84.2)	0.962	0.728–1.273		64 (88.9)	1.016	0.772–1.337	
CVA				0.063				0.033^∗^
Yes	1 (33.3)	0.206	0.078–0.544		1 (33.3)	0.138	0.048–0.402	
No	69 (86.3)	2.588	0.521–12.851		69 (90.8)	2.724	0.549–13.516	
Liver disease				1.000				0.523
Yes	5 (83.3)	0.923	0.143–5.950		5 (83.3)	0.649	0.097–4.359	
No	66 (84.6)	1.015	0.701–1.470		66 (89.2)	1.070	0.742–1.544	
PUD				1.000				1.000
Yes	18 (85.7)	1.019	0.830–1.251		18 (90)	1.019	0.857–1.211	
No	53 (84.1)	0.900	0.273–2.964		53 (88.3)	0.857	0.194–3.795	

CI: confidence interval; BMI: body mass index; NSAIDs: nonsteroidal anti-inflammatory drugs; HTN: hypertension; DM: diabetes mellitus; CVA: cerebrovascular accident; PUD: peptic ulcer disease. ^∗^*P* < 0.05.

**Table 4:** Risk factor associated with eradication failure by multiple logistic regression analysis.

Variables	Odds ratio	95% CI	*p* value
≥60 years old	1.036	0.124–8.628	0.974
Female	0.394	0.033–4.758	0.464
Overweight BMI	5.929	1.047–33.585	0.044^∗^
HTN	0.040	0.001-2.893	0.140
DM	11.968	0.091–1569.267	0.318
CVA	18.571	1.488–231.719	0.023^∗^
Liver disease	17.027	0.374–774.174	0.145
Without peptic ulcer	1.262	0.089–17.928	0.864

CI: confidence interval; BMI: body mass index; HTN: hypertension; DM: diabetes mellitus; CVA: cerebrovascular accident. ^∗^*P* < 0.05.

**Table 5:** Adverse reactions.

	*n* (%)
All	8 (10%)
Nausea/vomiting	3
Dizziness	2
Abdominal pain	1
Diarrhea	1
Headache	1
